# Disease knowledge and medication adherence among rheumatoid arthritis patients receiving pharmacist-led education: prospective single-arm interventional study

**DOI:** 10.3389/fphar.2026.1813901

**Published:** 2026-05-29

**Authors:** Shaimaa Alsukkar, Abdullah Burak Karaduman, Elif Aras-Atik, Kamer Tecen

**Affiliations:** 1 Department of Clinical Pharmacy, Faculty of Pharmacy, Anadolu University, Eskişehir, Türkiye; 2 Department of Pharmaceutical Toxicology, Faculty of Pharmacy, Anadolu University, Eskişehir, Türkiye; 3 Department of Clinical Pharmacy, Faculty of Pharmacy, Atatürk University, Erzurum, Türkiye

**Keywords:** clinical pharmacy, disease knowledge, medication adherence, patient education, rheumatoid arthritis

## Abstract

**Background:**

Pharmacists, with their pivotal roles and responsibilities in health sector, play a vital role in improving patients’ knowledge and medication adherence. This study aimed to assess the effect of pharmacist-led educational interventions on disease knowledge and medication adherence in patients with rheumatoid arthritis (RA).

**Methods:**

The prospective single-arm interventional study was conducted at a community pharmacy between 1 May 2025 and 31 August 2025. The data on knowledge about RA (disease knowledge, pharmacological treatments, non-pharmacological treatments, comorbidity, self-care and adaptive skills) and medication adherence were collected from RA patients at baseline and follow-up at 12 weeks. Factors related to disease knowledge and medication adherence were evaluated. The Rheumatoid Arthritis Knowledge Level Survey (RAKE) and the Compliance Questionnaire for Rheumatology (CQR) were administered to measure knowledge about RA and medication adherence, respectively.

**Results:**

A total of 95 patients were included in the study. After the pharmacist-led educational interventions, more patients responded correctly to domain disease knowledge (<0.001), pharmacological treatments (<0.001), non-pharmacological treatments (<0.001), comorbidity (<0.001), and self-care (<0.001). A statistically significant increase was observed in the total RAKE score (46.16 ± 16.16 vs. 72.96 ± 14.40; p < 0.001). When the total CQR scores before and after the education were compared, a significant increase in medication adherence was observed after the education (60.44 ± 10.35 vs. 66.11 ± 9.42; p < 0.001 respectively). The study identified a significant correlation between disease knowledge level and female gender (before the education: r = 0.344, p = 0.0011; after the education: r = 0.231, p = 0.02). Additionally, it has been observed that medication adherence was higher in individuals with a prolonged duration of disease (after the education: r = 0.235, p = 0.005) and in older adults (before the education: r = 0.384, p < 0.001; after the education: r = 0.310, p = 0.002).

**Conclusion:**

Rheumatoid arthritis patients’ disease knowledge and medication adherence improved after the clinical pharmacist’s intervention. The study recommends that it is necessary to conduct larger and multi-centered studies with longer follow-up durations so that sustainability and long-term effects of pharmacist-led interventions can be examined in detail. The female participants were found to possess a high level of disease knowledge in the study. Older individuals and people with long-term diseases had higher drug adherence. Further studies should be conducted with a large number of patients with rheumatoid arthritis to enhance generalizability to a wider population.

## Introduction

Rheumatoid Arthritis (RA) is a persistent inflammatory condition mostly impacting the joints, leading to pain, swelling, and reduced mobility. The ailment results in joint malformation and impairment over time (Radu and Bungau, 2021). The condition is the third primary cause of disability, following osteoarthritis and gout, and affects approximately 1% of the global population. Impaired mobility leads to diminished productivity in patients and exacerbates their quality of life (Smolen et al., 2018). While medication intervention is important for managing acute flares and episodic pain caused by the disease, non-pharmacologic treatments are also significant (Grygiel-Górniak et al., 2025).

International guidelines (Singh et al., 2016; Sm et al., 2020) indicate that treatment decisions in RA should involve a shared decision-making process between patients and physicians, taking the patient’s values, preferences, and comorbidities into account. Patient education aims to provide patients and their families with the skills required to effectively manage their disease. The European Alliance of Associations for Rheumatology (EULAR) promotes patient education as a fundamental component of standard care for individuals with inflammatory arthritis, enabling the development of self-care and coping skills. Patient education encompasses various activities structured around a planned interactive process, which may take place in group sessions or face-to-face, as well as online formats, tailored to meet patients’ needs and values (Zangi et al., 2015).

Medication adherence in RA patients is frequently inadequate, varying between 30% and 80%, underscoring the necessity for efforts to enhance adherence (Voora et al., 2020). These factors increase the need for pharmacists, who play a crucial role in overseeing drug therapy, managing drug information, providing appropriate counseling, identifying adverse drug reactions (ADRs) and drug interactions, and developing suitable interventions that ultimately lead to more effective therapeutic outcomes. Numerous studies have demonstrated the beneficial effect of pharmacist-led interventions on patient clinical outcomes (Ozdemir et al., 2023; Kara et al., 2021; Bahap et al., 2021). Pharmacist-led educational interventions help patients develop awareness about the signs and symptoms of RA, create ways to minimize its negative consequences, improve their treatment choices, and promote drug adherence, ultimately leading to better health and improved quality of life (Naqvi et al., 2019; Khadka et al., 2025; Taibanguay et al., 2019).

In Türkiye, RA prevalence for the general population was calculated as 0.56%; 0.10% for males and 0.89% for females (Tuncer et al., 2018). Insufficient studies exist regarding the understanding of the disease and medication adherence among RA patients in Türkiye. This study aims to determine the knowledge levels of RA patients in Türkiye regarding their disease and treatment, their medication adherence, and to determine the impact of pharmacist-led educational interventions on improving these levels.

## Materials and methods

### Study design and setting

This study was designed as a prospective single-arm interventional study. In this design, all participants receive the same intervention and are tracked for a certain time to evaluate predefined outcomes. Since there is no concurrent control group, outcomes are typically compared with baseline measurements or historical data (Wang et al., 2024). The study was conducted at a community pharmacy in Istanbul between 1 May 2025 and 31 August 2025. In the study, RA knowledge level (disease knowledge, pharmacological treatments, non-pharmacological treatments, comorbidity, self-care, and adaptive skills) and RA patients’ medication adherence were determined in a community pharmacy by a clinical pharmacist working at this pharmacy. The primary outcome of this study was to evaluate the effect of the clinical pharmacist-led educational intervention on patients’ rheumatoid arthritis knowledge, as measured by the Rheumatoid Arthritis Knowledge Level Survey - Turkish Version (RAKE-T) total score. Secondary outcomes included the assessment of medication adherence using the Compliance Questionnaire for Rheumatology - Turkish Version (CQR-T) and the evaluation of factors related to rheumatoid arthritis knowledge and medication adherence. The study was approved by the ethics committee (No: 878425). The participants completed a written informed consent form.

### Study population

The study included patients over 18 who had been diagnosed with RA for more than 1 year, had already started RA treatment, were open to communication, and provided written consent.

The sample size calculation was performed using the G*Power 3.1 software to strengthen the statistical power of the study. As suggested in the related studies in the literature (Pehlivan et al., 2024), an effect size of 0.35 was assumed in the primary outcome with rheumatoid arthritis knowledge level.

For the analysis, the significance level (α) was set to 0.05, and a one-tailed test was applied. The statistical power (1-β) was determined as 0.95. Based on these parameters, the required total sample size was calculated to be 90. This sample size ensures that the study has sufficient power to detect statistically significant results.

### Study instrument

#### The survey

The survey questions focus on collecting demographic data including gender, age, marital status, duration of rheumatoid arthritis, comorbidities, and medications used.

#### Rheumatoid arthritis knowledge level survey - Turkish version (RAKE-T)

The RAKE-T was developed by Rodère et al. to assess the patient knowledge in RA (Rodère et al., 2022). The survey includes a total of 45 RA-related items, covering disease information (10 items), pharmacological treatments (14 items), non-pharmacological treatments (7 items), comorbidity (1 item), self-care (5 items), and adherence skills (8 items). The items in the questionnaire are scored as “True” (1 point), “False” (0 points), and “Don’t know” (0 points), with incorrect statements scored in reverse. The total score from the surveys is calculated by converting it to 100 points: (total score x 100)/45. According to this formula, the score ranges from 0 to 100. The increasing score indicates a high level of knowledge. The Turkish validation of the survey was conducted by Pehlivan et al. (2024).

#### Compliance questionnaire for rheumatology - Turkish version (CQR-T)

The Compliance Questionnaire for Rheumatology (CQR) is a self-reported instrument developed to assess medication adherence in patients with rheumatic diseases. It evaluates patients’ beliefs, attitudes, and behaviors related to their prescribed antirheumatic treatments and is widely used to identify levels of treatment compliance in clinical research and practice de Klerk et al. (1999). The Turkish validation of the survey was conducted by Çinar et al. (2016). CQR-T is the only validated questionnaire for measuring treatment adherence in patients with rheumatic diseases. This 19-item survey measures patients’ agreement with specific statements using a four-point Likert scale, ranging from “strongly disagree” (1 point) to “strongly agree” (4 points). The result scores range from 0 (non-adherence) to 100 (perfect adherence). Patients are grouped as adherent and non-adherent using an 80% cutoff score, as suggested in the previous studies (Tolu et al., 2020; Tek et al., 2021).

#### The education brochure

As part of the intervention, a patient education program supported by an educational brochure was provided for patients with rheumatoid arthritis. The content of the education included information on the pathophysiology of rheumatoid arthritis, commonly prescribed antirheumatic medications, possible adverse effects, the importance of medication adherence, and principles of treatment management and regular clinical follow-up. The educational content was developed according to the current literature review and patient education principles (Singh et al., 2016; Sm et al., 2020). The education was delivered in a single session lasting approximately 20–30 min and was visually supported with a printed brochure prepared using clear and patient-friendly language. In addition to the standardized presentation made for all participants, the education was partially individualized by addressing patients’ questions and specific concerns regarding their medications and disease management.

### Study procedure

**FIGURE 1:**
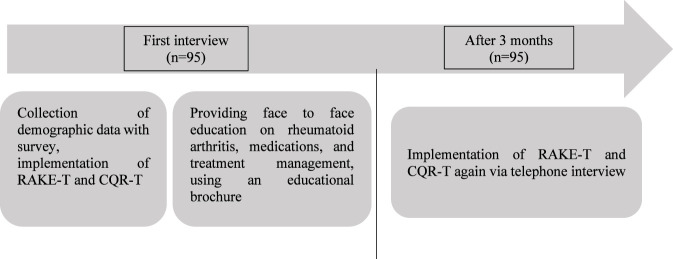
Design of the study.

All patients with rheumatoid arthritis meeting the inclusion criteria were consecutively included in the study. The clinical pharmacist conducted face-to-face interviews with these patients who visited the community pharmacy and agreed to participate in the study. The interviews were conducted in a private room at the pharmacy as one-on-one sessions, lasting approximately 20–30 min each. The clinical pharmacist first administered the survey questions to the patients to assess their demographic information. Afterward, the clinical pharmacist administered the “Rheumatoid Arthritis Knowledge Level Survey - Turkish Version (RAKE-T)” to determine the current level of rheumatoid arthritis knowledge and the “Compliance Questionnaire for Rheumatology - Turkish Version (CQR-T)” to evaluate medication adherence. Immediately after the initial survey (about demographic information), RAKE-T and CQR-T administration, the same participants received standardized education from a clinical pharmacist on rheumatoid arthritis, medications, and treatment management, which was supported by an educational brochure prepared based on the literature review (Singh et al., 2016; Sm et al., 2020). At the end of the first visit, the pharmacist noted down the patients’ phone numbers so that they could be used for a telephone interview 12 weeks later. Twelve weeks after the education, the participants were contacted by phone, and the RAKE-T and CQR-T surveys were administered again (Figure 1).

### Statistical analysis

We performed all the statistical analyses using Statistical Package for Social Sciences (SPSS) version 26. The variables were characterized by mean and standard deviation (SD), median and interquartile range (IQR), count, and percentage. The data’s normality was assessed using the Kolmogorov-Smirnov test. A dependent groups t-test was used to compare RAKE-T and CQR-T scores before and after the training. In addition to statistical significance testing, effect size was calculated using Cohen’s d. The McNemar-Bowker test was preferred to compare the variables with 3 or more categories. To evaluate the factors affecting RAKE-T and CQR-T scores, bivariate correlation analysis was performed for numerical data, and linear regression analysis for categorical data. P value <0.05 was considered statistically significant.

## Results

A total of 95 eligible patients were identified and approached for participation. None of the patients declined to participate, and all enrolled patients completed the 12-week follow-up without any loss to follow-up. The majority of these patients were male (n = 63, 66.3%). Demographic data are given in Table 1. The top three most used medications in rheumatoid arthritis treatment were conventional synthetic disease-modifying anti-rheumatic drugs (csDMARDs), nonsteroidal anti-inflammatory drug (NSAIDs), and analgesics, respectively.

**TABLE 1:** Demographic characteristics of the patients (n = 95).

Variables	Patients (n = 95)
Age, mean ± SD (years)	45.35 ± 15.31
Gender, n (%)	
Female	32 (33.7)
Male	63 (66.3)
Marital status, n (%)	
Married	21 (22.1)
Single	74 (77.9)
Disease duration, median [IQR] (years)	5 [IQR:10]
Current treatments NSAIDs, n (%)	64 (%71.9)
Diclofenac sodium	17 (%26.56)
Ibuprofen	14 (%21.88)
Nimesulide	7 (%10.94)
Dexketoprofen trometamol	12 (%18.75)
Naproxen	10 (%15.63)
Indomethacin	4 (%6.25)
Analgesics, n (%)	57 (%64.0)
Paracetamol	41 (%71.93)
Codeine-cafeine-parasetamol	16 (%28.07)
Glucocorticoids, n (%)	52 (%58.4)
Methylprednisolone	15 (%28.85)
Prednisolone	37 (71.15)
csDMARDs, n (%)	77 (%86.5)
Methotrexate	53 (%68.83)
Hydroxychloroquine	24 (%31.17)
bDMARDs, n (%)	12 (%13.5)
Tocilizumab	6 (%50.00)
Golimumab	5 (%41.67)
Adalimumab	1 (%8.33)
tsDMARDS, n (%)	1 (%1.1)
Baricitinib	1 (%100)

Categorical variables reported as frequency (percent, IQR: Interquartile range), SD: standard deviation.

bDMARDs: biological disease-modifying anti-rheumatic drugs, csDMARDs: conventional synthetic disease-modifying anti-rheumatic drugs, NSAIDs: nonsteroidal anti-inflammatory drug, tsDAMARs: targeted synthetic disease-modifying anti-rheumatic drugs.

**FIGURE 2:**
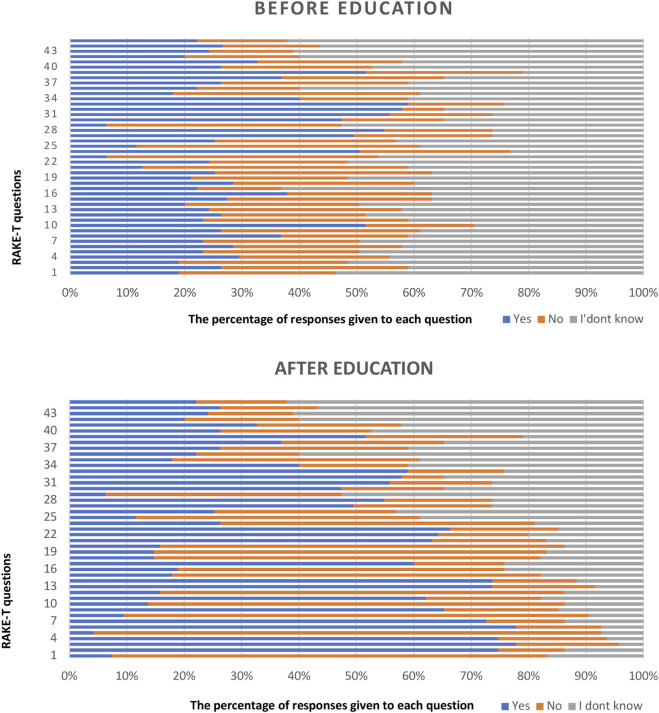
Distribution of RAKE-T responses before and after the clinical pharmacist’s educational intervention (n = 95).

In the responses provided for the RAKE-T questions prior to the clinical pharmacist’s educational intervention, the percentage of “I don't know” responses ranged between 21.1% (Q39, If you have rheumatoid arthritis, you should not exercise) and 62.1% (Q45, Recognition of disabled employee status often allows for workplace adjustments). There were no questions with a correct response rate above 50.0% (Figure 2).

After the clinical pharmacist’s educational intervention, the percentage of “I don’t know” responses to RAKE-T questions increased from 4.2% (Q3, Smoking increases the likelihood of developing rheumatoid arthritis) to 62.1% (Q45, Recognition of disabled employee status often allows for workplace adjustments). The patients answered over 50% of the 24 questions correctly. These questions and their correct answer rates are: 54.7% for Q24 (A special diet is necessary for rheumatoid arthritis, excluding certain types of food), 56.8% for Q16 (Cortisone is one of the disease-modifying drugs), %60.0 for Q17 (If there is blackening in the stool, non-cortisone anti-inflammatory drugs should be discontinued), %62.1 for Q11 (Rheumatoid arthritis disease-modifying antirheumatic drug treatments should be monitored due to potential side effects), 63.2% for Q21 (Physical activity/exercise helps reduce fatigue in rheumatoid arthritis), 64.2% for Q22 (Splints/orthoses can be helpful during disease flares) and Q15 (Long-term use of cortisone can be stopped abruptly), 65.3% for Q9 (The goal of rheumatoid arthritis management is to achieve remission (reduction or elimination of disease symptoms), 66.3% for Q23 (Proper footwear can reduce pain and deformities), 67.4% for Q18 (The only pain reliever allowed for rheumatoid arthritis is acetaminophen/paracetamol), 68.4% for Q19 (When taking painkillers (analgesics), you should stop taking anti-inflammatory medications), 70.5% for Q12 (Treatment for rheumatoid arthritis is the same for all patients), 72.6% for Q10 (Corticosteroids or non-steroidal anti-inflammatory drugs are sufficient to treat rheumatoid arthritis), 73.7% for Q13 (If an infection or fever is present, biological drug treatment should be interrupted) and Q14 (Biological drug treatment should be discontinued before a planned surgery), 74.7% for Q2 (Rheumatoid arthritis is an autoimmune disease) and Q4 (Joint swelling can be a sign of rheumatoid arthritis inflammation), 75.8% for Q1 (Rheumatoid arthritis is passed directly from parent to child), 77.9% for Q3 (Smoking increases the likelihood of developing rheumatoid arthritis) and Q6 (Rheumatoid arthritis can cause fatigue), 81.1% for Q8 (A blood test is sufficient to diagnose rheumatoid arthritis) and 88.4% for Q5 (Rheumatoid arthritis pain only occurs during the day) (Figure 2).

The subcategory scores obtained in the RAKE-T questionnaire and the total scores before and after the clinical pharmacist’s educational intervention are shown in Table 2. After the education, more patients responded correctly to domain disease knowledge (<0.001), pharmacological treatments (<0.001), non-pharmacological treatments (<0.001), comorbidity (<0.001) and self-care (<0.001). A statistically significant increase was observed in the total RAKE-T score (46.16 ± 16.16 vs. 72.96 ± 14.40; (<0.001) after the education.

**TABLE 2:** RAKE-T score by domain before and after the education (n = 95).

Domains	Before the education Mean (±SD)	After the education Mean (±SD)	*p* value	t	Cohen’s d
Disease knowledge (Q1-8, Q35-37)	4.41 (2.07)	7.77 (1.80)	<0.001	−17.012	1.75
Pharmacological treatments (Q9–19, Q35–37)	5.56 (3.05)	9.54 (2.35	<0.001	−17.597	2.78
Non-pharmacological treatments (Q20–24, Q38–39)	2.15 (1.46)	5.02 (1.42)	<0.001	−20.558	3.25
Comorbidity (Q25)	0.43 (0.49)	0.69 (0.46)	<0.001	−5.794	0.92
Self-care (Q26–28, Q40–41)	1.63 (1.14)	3.50 (1.21)	<0.001	−14.065	2.22
Adaptive skills (Q29–32, Q42–45)	6.60 (2.17)	6.28 (1.67)	0.19	1.311	0.21
Total RAKE-T score	46.16 (16.66)	72.96 (14.40)	<0.001	9.714	1.54

Score 0–100, SD means standard deviation, The distributions are normal, t-test was performed. Effect sizes were calculated using Cohen’s d.

The analyses of the responses of patients to the CQR-T questionnaire revealed statistically significant differences in all the items except for 5, 6, 7, and 17 after the education. When the total CQR-T scores before and after education were compared, a statistically significant increase in medication adherence was observed after the education (60.44 ± 10.35 vs. 66.11 ± 9.42; p < 0.001) (Table 3). According to CQR-T results, when adherence was considered to be ≥ 80 points, no significant difference was found between pre- and post-education comparisons (p < 0.05) (before education: perfect adherence = 3 patients, non-adherence = 92 patients; after education: perfect adherence = 3 patients, non-adherence = 92 patients).

**TABLE 3:** Responses to the CQR-T before and after education (n = 95).

Items of CQR-T		Before education Mean (±SD)	After education Mean (±SD)	t	*Cohens’d*	*p* value
1.	If the rheumatologist tells me to take the medicines, I do so	3.23 (±0.51)	3.33 (0,55)	2.764	0.44	0.007
2.	I Take my anti-rheumatic medicines because I, then, have fewer problems	3.09 (0.63)	3.23 (0.58)	2.481	0.39	0.01
3.	I Definitely don’t dare to miss my anti-rheumatic medications	2.43 (0.90)	2.80 (0.99)	4.158	0.66	<0.001
4.	If I can help myself with alternative therapies, I prefer that to what my rheumatologist prescribes.	2.35 (0.89)	2.87 (0.80)	5.459	0.86	<0.001
5.	My medicines are always stored in the same place, and that’s why I don’t forget them	3.34 (0.61)	3.28 (0.59)	1.924	0.30	0.05
6.	I Take my medicines because I have complete confidence in my rheumatologist	3.14 (0.54)	3.13 (0.51)	0.228	0.04	0.82
7.	The most important reason to take my anti-rheumatic medicines is that I can still do what I want to do	3.06 (0.61)	3.01 (0.61)	1.216	0.19	0.22
8.	I don’t like to take medicines. If I can do without them, I will	1.81 (0.82)	2.36 (1.13)	4.907	0.78	<0.001
9.	When I am on vacation, it sometimes happens that I don’t take my medicines	2.73 (0.81)	2.92 (0.68)	2.569	0.41	0.01
10.	I Take my anti-rheumatic drugs, for otherwise what’s the point of consulting a rheumatologist?	3.08 (0.64)	2.70 (0.92)	4.269	0.67	<0.001
11.	I don’t expect miracles from my anti-rheumatic medicines	2.24 (0.73)	2.82 (0.92)	6.228	0.98	<0.001
12.	If you can’t stand the medicines you might say: “throw it away, no matter what	2.53 (0.69)	2.78 (0.66)	4.098	0.65	<0.001
13.	If I don’t take my anti-rheumatic medicines regularly, the inflammation returns	2.90 (0.60)	2.81 (0.67)	2.102	0.33	0.03
14.	If I don’t take my anti-rheumatic medicines, my body warns me	2.96 (0.69)	3.09 (0.78)	2.162	0.34	0.03
15.	My health goes above everything else and if I have to take medicines to keep well, I will	3.40 (0.49)	3.47 (0.50)	2150	0.34	0.03
16.	I Use a dose organizer for my medications	2.49 (0.69)	3.02 (0.75)	5.874	0.93	<0.001
17.	What the doctor tells me, I hang on to	3.01 (0.61)	2.97 (0.60)	0.686	0.11	0.49
18.	If I don’t take my anti-rheumatic medicines, I have more complaints	2.98 (0.75)	3.17 (0.54)	2.423	0.38	0.01
19.	It happens every now and then, I go out for the weekend and then I don’t take my medicines	2.64 (0.75)	2.93 (0.66)	3.73	0.59	<0.001
	Total CQR-T	60.44 (±10.35)	66.11 (±9.42)	7.935	1.25	p < 0.001

t-test was performed. Effect sizes were calculated using Cohen’s d.

The study examined the factors affecting the total RAKE-T and CQR-T scores before and after the education. A statistically significant positive relationship was found between female gender and the RAKE-T score (before the education: r = 0.344, p = 0.0011; after the education: r = 0.231, p = 0.02). There was not any significant relationship between age, marital status, duration of illness, and total RAKE-T score (p > 0.05).

Only age (before the education: r = 0.384, p < 0.001 and after the education: r = 0.310, p = 0.002) and duration of illness (after education: r = 0.235, p = 0.005) showed a positive statistically significant correlation with the CQR-T score. No statistically significant relationship was found between gender, marital status, and medications used, and the total CQR-T score (p > 0.05).

## Discussion

The study assessed the impact of educational intervention provided by pharmacists on disease knowledge and medication adherence among rheumatoid arthritis patients. Our study revealed that rheumatoid arthritis patients’ disease knowledge and medication adherence improved following the clinical pharmacist’s intervention.

In the responses to the RAKE-T questions before the clinical pharmacist’s educational intervention, there weren’t any questions with a correct response rate above 50% and the mean total RAKE-T score was 46.16 ± 16.16 in this study. In the study by Rodere et al., the mean total RAKE score was 66.8 ± 16.4. Six questions had a >50% rate of correct responses (Rodère et al., 2022). In a study conducted in India, the mean total RAKE score was found to be 54.88 ± 12.11. Twenty-six questions showed a correct response rate above 50% (Juneja et al., 2025). This study revealed a lower level of knowledge among patients regarding RA disease and its treatment when compared to previous studies. Several factors may explain this relatively lower level of knowledge observed in our patient population. Sociocultural characteristics, including differences in educational background, health literacy, and access to reliable medical information may play a significant role. Patients typically depend on physicians for information regarding their diseases and its management. Nonetheless, patients might have limited opportunities for education due to the heavy workloads of physicians. In addition, cultural perceptions of the disease, traditional beliefs about the treatment methods, and limited awareness of the long-term nature of RA may influence patients’ understanding of the disease and its management. Socioeconomic barriers and restricted access to educational resources may further contribute to this knowledge gap, making it difficult for patients to receive comprehensive information about rheumatoid arthritis and its management options. This gap in understanding clearly indicates that there must be improved educational resources and outreach programs. By enhancing patient education, we can encourage individuals to take a more active role in managing their conditions.

In the study by Rodere et al., the participant patients showed higher progression comorbidity, non-pharmacological treatments, and self-care after the educational intervention (Rodère et al., 2022). In this study, after the clinical pharmacist’s educational intervention, high progress was observed in all subgroups of RAKE, and there was a statistically significant increase in all subgroups (p < 0.001) except for adaptive skills (p = 0.19). Similar to this study, many studies have found that patients with rheumatoid arthritis achieve significant improvements in disease and treatment knowledge following the educational intervention of a clinical pharmacist (Naqvi et al., 2019; Khadka et al., 2025; Bottois et al., 2025). Several interrelated mechanisms may contribute to the positive impact of pharmacist-led education. Pharmacists have extensive expertise in pharmacotherapy and are uniquely positioned to provide comprehensive counseling on medication use, potential adverse effects, and the long-term benefits of adherence. By addressing patients’ concerns and misconceptions about antirheumatic medications, pharmacist-led interventions may help eliminate negative medication beliefs and stimulate patients’ confidence in their treatment. Furthermore, the involvement of pharmacists in rheumatology care teams may enhance multidisciplinary collaboration and promote more patient-centered care. From a health system viewpoint, including pharmacists in regular rheumatology care can result in more effective management of medications, improve treatment results, and possibly lower the need for healthcare services due to poorly managed diseases.

In this study, the mean CQR score before the clinical pharmacist’s educational intervention was 60.44 (standard deviation: 10.3). Similar to our study, the mean CQR score in the study conducted in Denmark (Knudsen and de Thurah, 2023) and Korea (Lee et al., 2021) was about 60. In a study conducted with patients with RA, polymyalgia rheumatica (PMR), and gout, had a higher CQR score than our study, the mean CQR score was 76.6 (standard deviation 12.8) (de Klerk et al., 2023). In another study examining adherence to 1-year methotrexate treatment in rheumatoid arthritis patients, the median CQR score was 70.1–70.6 (de Thurah et al., 2010).

However, when adherence was defined using the ≥80% cut-off value, no statistically significant difference was observed in the proportion of adherent patients in this study. This apparent discrepancy may be explained by the loss of information that occurs when continuous variables are dichotomized (Altman and Royston, 2006). These findings suggest that the educational intervention produced a clinically meaningful improvement in adherence behavior even though it did not significantly increase the proportion of patients exceeding the adherence cut-off.

A statistically significant improvement in medication adherence was observed after the clinical pharmacist’s educational intervention (60.44 ± 10.35 vs. 66.11 ± 9.42; p < 0.001). This aligns with findings from various studies that have demonstrated improved disease and medication knowledge, as well as medication adherence, when a pharmacist supports their role in assessing autoimmune disorders (Gutermann et al., 2021; Anghel et al., 2018; Oliveira-Santos et al., 2019; Sah et al., 2021). A remarkable improvement in patient medication adherence was evidenced by a randomized controlled study conducted in Thailand, which demonstrated that patient education increased the adherence rate from 92.21 ± 14.05 to 97.59 ± 10.07 (P = 0.002) in the intervention group (Taibanguay et al., 2019). A conflicting finding regarding medication adherence was found in a single-center randomized controlled trial involving 123 RA patients in the Netherlands, which indicated that a motivational interviewing program did not result in a significant change in medication adherence among RA patients (Zwikker et al., 2014). One reason for this could be the improvement in belief and compliance, which may have already changed positively before the actual intervention took place. The improvement may be due to ‘regression to the mean’ (Twisk and De Vente, 2008), the Hawthorne effect (McNicho et al., 2012) phenomenon, or the acceptance meeting held by the researchers at the beginning of the study (Gamble et al., 2011). Another reason for this, as noted by the researchers, is that although a good level of patient-centeredness was achieved, the overall treatment integrity level of the intervention was below the optimal level. This issue could also affect the study’s results. Additionally, the patients included in the study had a long disease duration (average: >14 years). Changing existing beliefs and adherence behaviors in patients with such a long disease duration may not be easy, as they may have deeply ingrained habits and perspectives about their treatment and health management that are resistant to change (Willey et al., 2020). All these reasons may cause the motivational interviewing program to be insufficient in increasing medication adherence in patients, particularly because patients with long disease durations may have deeply-rooted beliefs and habits that are less likely to change.

In the present study, medication adherence among patients with rheumatoid arthritis significantly improved following the education provided by a clinical pharmacist. This finding is clinically relevant because contemporary rheumatoid arthritis management is based on a treat-to-target strategy, in which sustained and consistent use of disease-modifying antirheumatic drugs (DMARDs) is essential to achieve remission or, at least, low disease activity (Sm et al., 2020; Fraenkel et al., 2021). However, medication adherence in rheumatoid arthritis remains suboptimal in real-world settings, as some previous studies report that a considerable proportion of patients do not use their medications as prescribed, thereby compromising treatment effectiveness (Pasma et al., 2013; Scheiman-Elazary et al., 2016). Significantly, even modest enhancements in adherence may yield substantial clinical consequences. Studies have reported that higher adherence to DMARD therapy is associated with better disease control, lower disease activity scores, and an increased likelihood of achieving remission. Also, taking medication regularly helps keep drug levels high and stops disease flares, which in the end leads to better long-term functional outcomes and quality of life (Aksoy et al., 2024). In this situation, programs led by pharmacists could be an efficient way to boost adherence by teaching patients more about their disease and medications, providing solutions for any concerns about their medications, and emphasizing the need to take their treatment regularly.

In this study, a significant association was found between patients’ level of knowledge about rheumatoid arthritis and female gender, but no association was detected with age, marital status, disease duration, or the type of medication used. Additionally, medication adherence was found to be higher in elderly patients and those who have a long duration of illness. Previous studies focusing on the sociodemographic factors associated with medication adherence and patients’ level of knowledge about disease have yielded inconsistent findings. Hennel and Neame found women to be more knowledgeable about the disease, which is consistent with the related finding of our study (Hennell et al., 2004; Neame et al., 2005); however, no difference between genders was reported in the study by Tasci-Bozbas et al. (2018). Verstappen observed that patients with extended disease duration demonstrated better knowledge of their condition (Verstappen, 2015). Previous studies, however, indicated no correlation between disease knowledge and disease duration (Salman et al., 2014; Kamruzzaman et al., 2020). Albiss et al. reported a significant relationship between participants’ knowledge of rheumatoid arthritis and age (Albiss et al., 2025), whereas another study did not find such a relationship (Kamruzzaman et al., 2020). Gender differences did not reach statistical significance, as previously demonstrated (Townsend et al., 2014). Other studies, including those by Townsend et al., Sokka et al., and Intriago et al., demonstrate that women typically seek more information regarding their health conditions when compared to men (Townsend et al., 2014; Sokka et al., 2009; Intriago et al., 2019). Some studies reported that older age and male gender are associated with higher medication adherence among patients (Chu et al., 2015; Tkacz et al., 2014). In contrast, others have identified non-significant relationships between age and gender in regards to adherence (Calvo-Alén et al., 2017). Xia et al. found that disease duration was not associated with medication adherence (Xia et al., 2016). However, other researchers have reported that disease duration were associated with medication adherence (Rauscher et al., 2015). Considering that rheumatoid arthritis predominantly affects women, further research is necessary, particularly utilizing prospective study designs, to investigate the psychosocial factors contributing to lower therapy adherence among women when compared to men. This should include an examination of other relevant characteristics, such as disease severity, comorbidities, and perceived treatment efficacy, which may influence adherence.

### Strengths and weaknesses

This study presents certain limitations. First, the study conducted at a single center and results may not be generalizable to other setting. Comprehensive further studies involving multiple pharmacies from both rural and urban areas are necessary. Second, the study included a limited number of RA patients; nonetheless, the number of the participants satisfied the sample size criteria. Another limitation of this study is the absence of a control or comparison group. Improvements in medication knowledge and adherence occurred after the pharmacist-led educational intervention; however, we cannot credit the intervention alone for these results. Participation in the study and repeated exposure to the questionnaire may have increased patient awareness and contributed to improved responses, which may reflect a potential learning effect. Furthermore, the Hawthorne effect may have led participants to alter their behavior simply due to observation. Therefore, the findings should be interpreted with caution, and future studies including randomized controlled or comparative designs are warranted to better determine the causal impact of pharmacist-led educational interventions on medication adherence and knowledge in patients with rheumatoid arthritis. Fourth, a 12-week follow-up period may not be sufficient for evaluation. Long-term follow-up studies are necessary to assess the long-term impact of pharmacist interventions on medication adherence and disease knowledge. Fifth, different modes of data collection were used: baseline RAKE-T and CQR-T assessments were conducted face-to-face, while follow-up assessments were performed via telephone. Although telephone administration of structured patient-reported measures has been shown to be feasible and reliable in chronic diseases, and the CQR has previously been applied via telephone, there is no specific validation study for the telephone administration of the RAKE-T. Therefore, the difference in administration modes may have influenced patient responses. Lastly, the analysis included a limited number of demographic characteristics, including age, gender, marital status, disease duration, and medication type. We expect that additional demographic, psychosocial, or health-related characteristics—beyond those obtainable from retrospective pharmacy claims data—are linked to patient adherence and patients’ level of knowledge about rheumatoid arthritis. Further research is essential to clarify and predict patient characteristics linked to treatment behaviors and health outcomes. Furthermore, further research is required to investigate the impact of alterations in inflammatory markers and other clinical evaluations on patient adherence, persistence, and modifications in therapy.

Despite the limitations, the study also has strengths. First, the intervention was conducted through individual face-to-face consultations between the clinical pharmacist and each patient, which allowed a detailed assessment of medication adherence and disease-related knowledge. It is possible that such a direct interaction improved the reliability of patient responses and enabled the pharmacist to identify individual knowledge gaps and adherence barriers more accurately.

Second, the study employed a structured educational intervention delivered immediately after the baseline assessment. Providing education based on the patients’ initially identified needs allowed the intervention to be tailored to individual patients, which may enhance the effectiveness of pharmacist-led counseling and improve patient engagement. Third, the study included a prospective follow-up evaluation after 3 months with the same patient cohort, during which the same adherence and knowledge questionnaires were administered. This design allowed for the assessment of changes over time within the same individuals, thereby minimizing inter-individual variability and strengthening the internal validity of the findings. Another strength is the use of the same standardized questionnaires both before and after the intervention, ensuring methodological consistency and allowing reliable comparison of outcomes.

## Conclusion

Improvements in disease knowledge and medication adherence were observed among patients with rheumatoid arthritis following the clinical pharmacist–led educational intervention. These findings suggest that pharmacist-led education may play a supportive role in improving disease management. Additionally, the study emphasizes the need for future research involving larger, multi-center populations and longer follow-up periods to assess the sustainability and long-term effects of pharmacist-led interventions.

The study identified a significant relationship between patients’ knowledge level regarding rheumatoid arthritis and female gender. Moreover, it has been observed that medication adherence is higher in individuals with a prolonged duration of disease and in older adults. Further studies should be conducted with a large number of patients with rheumatoid arthritis so that the findings can be generalized to become generalizable to a broader population.

## Data Availability

The original contributions presented in the study are included in the article/supplementary material, further inquiries can be directed to the corresponding author.

## References

[B1] Aksoy N. Ozturk N. Agh T. Kardas P. (2024). Adherence to the antirheumatic drugs: a systematic review and meta-analysis. Front. in Medicine 11, 1456251. 10.3389/fmed.2024.1456251 39328321 PMC11424425

[B2] Albiss L. Muflih S. Hijazi B. Alshogran O. Y. Al-Querem W. Khurmah M. A. (2025). Disease knowledge and quality of life among rheumatoid arthritis patients: A cross-sectional study. BMC Rheumatol. 9, 77. 10.1186/s41927-025-00523-w 40597453 PMC12220810

[B3] Altman D. G. Royston P. (2006). The cost of dichotomising continuous variables. BMJ 332 (7549), 1080. 10.1136/bmj.332.7549.1080 16675816 PMC1458573

[B4] Anghel L. A. Farcaş A. M. Oprean R. N. (2018). Medication adherence and persistence in patients with autoimmune rheumatic diseases: A narrative review. Patient Prefer. Adherence. 12, 1151–1166. 10.2147/PPA.S165101 30013327 PMC6037147

[B5] Bahap M. Kara E. Cagla Sonmezer M. Inkaya A. C. Aydin‐Hakli D. Unal S. (2021). Pharmacist intervention to improve patients’ knowledge and attitude towards hepatitis B infection. Int. J. Clin. Pract. 75, e13952. 10.1111/ijcp.13952 33342028

[B6] Bottois C. Lopez Medina C. Dumas S. Hubert J. Belo S. Roux C. (2025). Impact of a clinical pharmacist consultation on enhancing knowledge and safety skills in patients with chronic inflammatory arthritis treated with bDMARDs. Clin. Exp. Rheumatol. 43, 1218–1226. 10.55563/clinexprheumatol/mhtjub 40371562

[B7] Calvo-Alén J. Monteagudo I. Salvador G. Vázquez-Rodríguez T. R. Tovar-Beltrán J. V. Vela P. (2017). Non-adherence to subcutaneous biological medication in patients with rheumatoid arthritis: A multicentre, non-interventional study. Clin. Exp. Rheumatol. 35, 423–430. 28032846

[B8] Chu L. H. Kawatkar A. A. Gabriel S. E. (2015). Medication adherence and attrition to biologic treatment in rheumatoid arthritis patients. Clin. Ther. 37, 660–668. 10.1016/j.clinthera.2014.10.022 25618317

[B9] Cinar F. I. Cinar M. Yilmaz S. Acikel C. Erdem H. Pay S. (2016). Cross-cultural adaptation, reliability, and validity of the Turkish version of the compliance questionnaire on rheumatology in patients with behçet’s disease. J. Transcult. Nurs. 27, 480–486. 10.1177/1043659615577699 25801762

[B10] de Klerk E. van der Heijde D. van der Tempel H. van der Linden S. (1999). Development of a questionnaire to investigate patient compliance with antirheumatic drug therapy. J. Rheumatol. 26, 2635–2641. 10606375

[B11] de Klerk E. van der Heijde D. Landewé R. van der Tempel H. van der Linden S. (2023). The compliance-questionnaire-rheumatology compared with electronic medication monitoring: A validation study. J. Rheumatol. 30, 2469–2475. 14677194

[B12] de Thurah A. Nørgaard M. Harder I. Stengaard-Pedersen K. (2010). Compliance with methotrexate treatment in patients with rheumatoid arthritis: influence of patients’ beliefs about the medicine. A prospective cohort study. Rheumatol. Int. 30, 1441–1448. 10.1007/s00296-009-1160-8 19823840

[B13] Fraenkel L. Bathon J. M. England B. R. St Clair E. W. Arayssi T. Carandang K. (2021). 2021 American college of Rheumatology guideline for the treatment of rheumatoid arthritis. Arthritis Care Res. Hob. 73 (7), 924–939. 10.1002/acr.24596 34101387 PMC9273041

[B14] Gamble J. Stevenson M. Heaney L. G. (2011). A study of a multi-level intervention to improve non-adherence in difficult to control asthma. Respir. Med. 105, 1308–1315. 10.1016/j.rmed.2011.03.019 21511454

[B15] Grygiel-Górniak B. Kowynia E. Abouzid M. Kwaśniewska A. Joks M. Majewska N. (2025). Quality of life in long-standing rheumatoid arthritis: what can help apart from treatment? A single-center cross-sectional observational study. J. Clin. Med. 14 (24), 8925. 10.3390/jcm14248925 41464826 PMC12733405

[B16] Gutermann L. Dumas S. Lopez-Medina C. Boissinot L. Cotteret C. Perut V. (2021). Impact of a pharmacist-led programme on biologics knowledge and adherence in patients with spondyloarthritis. Clin. Exp. Rheumatol. 39, 811–818. 10.55563/clinexprheumatol/pzc5lo 33124563

[B17] Hennell S. L. Brownsell C. Dawson J. K. (2004). Development, validation and use of a patient knowledge questionnaire (PKQ) for patients with early rheumatoid arthritis. Rheumatology. 43, 467–471. 10.1093/rheumatology/keh069 15024135

[B18] Intriago M. Maldonado G. Cárdenas J. Ríos C. (2019). Clinical characteristics in patients with rheumatoid arthritis: Differences between genders. Sci. World J. 2019, 8103812. 10.1155/2019/8103812 31354388 PMC6636593

[B19] Juneja S. Mittal M. Garg P. Somashekar M. Jain N. Duggal L. (2025). POS0413-HPR assessment of rheumatoid arthritis knowledge and its association with drug compliance and disease course in patients: a cross-sectional study using the rake questionnaire in A tertiary care centre in India. Ann. Rheum. Dis. 84, 650. 10.1016/j.ard.2025.05.799

[B20] Kamruzzaman A. K. M. Chowdhury M. R. Islam M. N. Sultan I. Ahmed S. Shahin A. (2020). The knowledge level of rheumatoid arthritis patients about their disease in a developing country: A study in 168 Bangladeshi RA patients. Clin. Rheumatol. 39, 1315–1323. 10.1007/s10067-019-04859-w 31828544

[B21] Kara E. Metan G. Bayraktar-Ekincioglu A. Gulmez D. Arikan-Akdagli S. Demirkazik F. (2021). Implementation of pharmacist-driven antifungal stewardship program in a tertiary care hospital. Antimicrob. Agents Chemother. 65, e0062921. 10.1128/AAC.00629-21 34152808 PMC8370214

[B22] Khadka S. Sankhi S. Marasine N. R. (2025). Pharmacist’s educational intervention on disease knowledge, medication adherence, and health-related quality of life among rheumatoid arthritis patients: a single centre, open-label, randomised controlled study. Hosp. Pharm. 60 (6), 00185787251337594. 10.1177/00185787251337594 40400907 PMC12089118

[B23] Knudsen L. R. de Thurah A. (2023). Face validity and reliability test of the Danish version of the compliance questionnaire rheumatology in patients with early rheumatoid arthritis. BMC Rheumatol. 7, 38. 10.1186/s41927-023-00364-5 37880764 PMC10598893

[B24] Lee J. Y. Lee S. Y. Hahn H. J. Son I. J. Hahn S. G. Lee E. B. (2021). Cultural adaptation of a compliance questionnaire for patients with rheumatoid arthritis to a Korean version. Korean J. Intern. Med. 26, 28–33. 10.3904/kjim.2011.26.1.28 21437159 PMC3056252

[B25] McNicholas N. Patel A. Chataway J. (2012). It is better to be in a clinical trial than not:lessons learnt from clinical neurology – the management of acute multiple sclerosis relapses. QJM 105, 775–780. 10.1093/qjmed/hcs070 22514268

[B26] Naqvi A. A. Hassali M. A. Naqvi S. B. S. Aftab M. T. (2019). Impact of pharmacist educational intervention on disease knowledge, rehabilitation and medication adherence, treatment-induced direct cost, health-related quality of life and satisfaction in patients with rheumatoid arthritis: Study protocol for a randomized controlled trial. Trials 20, 488. 10.1186/s13063-019-3540-z 31399128 PMC6688212

[B27] Neame R. Hammond A. Deighton C. (2005). Need for information and for involvement in decision making among patients with rheumatoid arthritis: A questionnaire survey. Arthritis Rheum. 53, 249–255. 10.1002/art.21071 15818715

[B28] Oliveira-Santos M. Verani J. F. Camacho L. A. de Andrade C. A. Klumb E. M. (2019). Effectiveness of pharmaceutical care for drug treatment adherence in women with lupus nephritis in Rio de Janeiro, Brazil: A randomized controlled trial. Lupus. 28, 1368–1377. 10.1177/0961203319877237 31558100

[B29] Ozdemir N. Aktas B. Y. Gulmez A. Inkaya A. C. Bayraktar-Ekincioglu A. Kilickap S. (2023). Impact of pharmacist-led educational intervention on pneumococcal vaccination rates in cancer patients: A randomized controlled study. Support. Care Cancer. 31, 194. 10.1007/s00520-023-07652-3 36856870 PMC9975445

[B30] Pasma A. van ’t Spijker A. Hazes J. M. W. Busschbach J. J. V. Luime J. J. (2013). Factors associated with adherence to pharmaceutical treatment for rheumatoid arthritis patients: a systematic review. Semin. Arthritis Rheum. 43 (1), 18–28. 10.1016/j.semarthrit.2012.12.001 23352247

[B31] Pehlivan S. Erbay Dallı Ö. Yılmaz Bozkurt Z. Ceyhan A. Pehlivan Y. (2024). Romatoid artrit bilgi düzeyi anketi türkçe versiyonunun (RAKE-T) psikometrik özelliklerinin değerlendirilmesi: Metodolojik bir çalışma. Uludağ Üniversitesi Tıp Fakültesi Derg. 50, 471–477. 10.32708/uutfd.1576774

[B32] Radu A. F. Bungau S. G. (2021). Management of rheumatoid arthritis: an overview. Cells 10 (11), 2857. 10.3390/cells10112857 34831081 PMC8616326

[B33] Rauscher V. Englbrecht M. van der Heijde D. Schett G. Hueber A. J. (2015). High degree of nonadherence to disease-modifying antirheumatic drugs in patients with rheumatoid arthritis. J. Rheumatol. 42, 386–390. 10.3899/jrheum.140982 25593229

[B34] Rodère M. Pereira B. Soubrier M. Fayet F. Piperno M. Pallot-Prades B. (2022). Development and validation of a self-administered questionnaire measuring essential knowledge in patients with rheumatoid arthritis. Rheumatol. Int. 42, 1785–1795. 10.1007/s00296-022-05090-8 35389078 PMC9439984

[B35] Sah S. K. Subramanian R. Ramesh M. Chand S. (2021). Impact of pharmacist care in the management of autoimmune disorders: A systematic review of randomized control trials and non-randomized studies. Res. Soc. Adm. Pharm. 17, 1532–1545. 10.1016/j.sapharm.2020.12.005 33423904

[B36] Salman S. Alnuaimi A. S. Lateef N. A. Kadhum R. (2014). Assessment of knowledge and attitude in a sample of patients with rheumatoid arthritis and its association with disease activity and severity: A cross-sectional study. Open J. Rheumatol. Autoimmune Dis. 4, 226. 10.4236/ojra.2014.44031

[B37] Scheiman-Elazary A. Duan L. Shourt C. Agrawal H. Ellashof D. Cameron-Hay M. (2016). The rate of adherence to antiarthritis medications and associated factors among patients with rheumatoid arthritis: a systematic literature review and metaanalysis. J. Rheumatology 43 (3), 512–523. 10.3899/jrheum.141371 26879354

[B38] Singh J. A. Saag K. G. Bridges S. L. Jr. Akl E. A. Bannuru R. R. Sullivan M. C. (2016). 2015 American college of rheumatology guideline for the treatment of rheumatoid arthritis. Arthritis Rheumatol. 68, 1–26. 10.1002/art.39480 26545940

[B39] Smolen J. S. Landewé R. M. Bijlsma J. W. Burmester G. R. Dougados M. Kerschbaumer A. (2020). EULAR recommendations for the management of rheumatoid arthritis with synthetic and biological disease-modifying antirheumatic drugs: 2019 update. Ann. Rheum. Dis. 79, 685–699. 10.1136/annrheumdis-2019-216655 31969328

[B40] Smolen J. S. Aletaha D. Barton A. Burmester G. R. Emery P. Firestein G. S. (2018). Rheumatoid arthritis. Nat. Rev. Dis. Prim. 4, 18001. 10.1038/nrdp.2018.1 29417936

[B41] Sokka T. Toloza S. Cutolo M. Kautiainen H. Makinen H. Gogus F. (2009). Women, men, and rheumatoid arthritis: Analyses of disease activity, disease characteristics, and treatments in the QUEST-RA study. Arthritis Res. Ther. 11, R7. 10.1186/ar2591 19144159 PMC2688237

[B42] Taibanguay N. Chaiamnuay S. Asavatanabodee P. Narongroeknawin P. (2019). Effect of patient education on medication adherence of patients with rheumatoid arthritis: randomized controlled trial. Patient Prefer. Adherence. 13, 119–129. 10.2147/PPA.S192008 30666095 PMC6333161

[B43] Tasci Bozbas G. Gurer G. (2018). The knowledge level of Turkish rheumatoid arthritis patients about their diseases. Anadolu Klin. 23, 1. 10.21673/anadoluklin.325831

[B44] Tekgoz E. Colak S. Çinar F. I. Yilmaz S. Çinar M. (2021). Non-adherence to colchicine treatment is a common misevaluation in familial Mediterranean fever. Turk. J. Med. Sci. 51, 2357–2363. 10.3906/sag-2102-328 33957721

[B45] Tkacz J. Ellis L. Bolge S. C. Meyer R. Brady B. L. Ruetsch C. (2014). Utilization and adherence patterns of subcutaneously administered anti-tumor necrosis factor treatment among rheumatoid arthritis patients. Clin. Ther. 36, 737–747. 10.1016/j.clinthera.2014.02.019 24661783

[B46] Tolu S. Rezvani A. Karacan I. Bugdayci D. Küçük H. C. Bucak O. F. (2020). Self-reported medication adherence in patients with ankylosing spondylitis: The role of illness perception and medication beliefs. Arch. Rheumatol. 35, 495–505. 10.46497/ArchRheumatol.2020.7732 33758806 PMC7945695

[B47] Townsend A. Backman C. L. Adam P. Li L. C. (2014). Women’s accounts of help-seeking in early rheumatoid arthritis from symptom onset to diagnosis. Chronic Illn. 10, 259–272. 10.1177/1742395314520769 24567194 PMC5760221

[B48] Tuncer T. Gilgil E. Kaçar C. Kurtaiş Y. Kutlay Ş. Bütün B. (2018). Prevalence of rheumatoid arthritis and spondyloarthritis in Turkey: A nationwide study. Arch. Rheumatol. 33, 128–136. 10.5606/ArchRheumatol.2018.6480 30207568 PMC6117145

[B49] Twisk J. W. R. De Vente W. (2008). The analysis of randomised controlled trial data with more than one follow-up measurement. A comparison between different approaches. Eur. J. Epidemiol. 23, 655–660. 10.1007/s10654-008-9279-6 18712484

[B50] Verstappen S. M. (2015). Rheumatoid arthritis and work: The impact of rheumatoid arthritis on absenteeism and presenteeism. Best. Pract. Res. Clin. Rheumatol. 29, 495–511. 10.1016/j.berh.2015.06.001 26612244

[B51] Voora L. Sah S. K. Bhandari R. Shastry C. S. Chand S. Rawal K. B. (2020). Doctor of pharmacy: Boon for healthcare system. Drug Invent. Today 14, 153–158.

[B52] Wang M. Ma H. Shi Y. Ni H. Qin C. Ji C. (2024). Single-arm clinical trials: design, ethics, principles. BMJ Support Palliat. Care 15 (1), 46–54. 10.1136/spcare-2024-004984 38834238 PMC11874317

[B53] Willey C. Redding C. Staflord J. Garfield F. Geletko S. Flanigan T. (2020). Stages of change for adherence with medication regimens for chronic disease: development and validation of a measure. Clin. Ther. 22, 858–871. 10.1016/s0149-2918(00)80058-2 10945512

[B54] Xia Y. Yin R. Fu T. Zhang L. Zhang Q. Guo G. (2016). Treatment adherence to disease-modifying antirheumatic drugs in Chinese patients with rheumatoid arthritis. Patient prefer. adherence. 10, 735–742. 10.2147/PPA.S98034 27217726 PMC4862390

[B55] Zangi H. A. Ndosi M. Adams J. Andersen L. Bode C. Boström C. (2015). EULAR recommendations for patient education for people with inflammatory arthritis. Ann. Rheum. Dis. 74, 954–962. 10.1136/annrheumdis-2014-206807 25735643

[B56] Zwikker H. E. van den Ende C. H. van Lankveld W. G. den Broeder A. A. den Hoogen F. H. van de Mosselaar B. (2014). Effectiveness of a group-based intervention to change medication beliefs and improve medication adherence in patients with rheumatoid arthritis: A randomized controlled trial. Patient Educ. Couns. 94, 356–361. 10.1016/j.pec.2013.12.002 24388126

